# *Croton lechleri* Extracts as Green Corrosion Inhibitors of Admiralty Brass in Hydrochloric Acid

**DOI:** 10.3390/molecules26247417

**Published:** 2021-12-07

**Authors:** Carlos Cevallos-Morillo, Pablo Cisneros-Pérez, Roxana Llive, Marvin Ricaurte, Carlos Reinoso, Miguel Angel Meneses, Maria del Cisne Guamán, Alex Palma-Cando

**Affiliations:** 1 Facultad de Ciencias Químicas, Universidad Central del Ecuador, Francisco Viteri s/n y Gato Sobral, Quito 170129, Ecuador; cacevallosm@uce.edu.ec; 2 Universidad Regional Amazónica Ikiam, Tena 150102, Ecuador; pablo.cisneros@ikiam.edu.ec (P.C.-P.); wendy.llive@ikiam.edu.ec (R.L.); 3 Grupo de Investigación Aplicada en Materiales y Procesos (GIAMP), School of Chemical Sciences and Engineering, Yachay Tech University, Hda. San José s/n y Proyecto Yachay, Urcuquí 100119, Ecuador; mricaurte@yachaytech.edu.ec; 4 School of Physical Sciences and Nanotechnology, Yachay Tech University, Hda. San José s/n y Proyecto Yachay, Urcuquí 100119, Ecuador; creinoso@yachaytech.edu.ec; 5 Departamento de Química y Ciencias Exactas, Universidad Técnica Particular de Loja, Loja 110150, Ecuador; mameneses@utpl.edu.ec (M.A.M.); mcguaman@utpl.edu.ec (M.d.C.G.)

**Keywords:** corrosion inhibition efficiency, admiralty brass, acid pickling solution, dragon’s blood, supercritical CO_2_ extraction, electrochemical impedance spectroscopy, Tafel plots, linear polarization resistance, XPS, SEM-EDS

## Abstract

*Croton lechleri,* commonly known as Dragon’s blood, is a tree cultivated in the northwest Amazon rainforest of Ecuador and Peru. This tree produces a deep red latex which is composed of different natural products such as phenolic compounds, alkaloids, and others. The chemical structures of these natural products found in *C. lechleri* latex are promising corrosion inhibitors of admiralty brass (AB), due to the number of heteroatoms and *π* structures. In this work, three different extracts of *C. lechleri* latex were obtained, characterized phytochemically, and employed as novel green corrosion inhibitors of AB. The corrosion inhibition efficiency (IE%) was determined in an aqueous 0.5 M HCl solution by potentiodynamic polarization (Tafel plots) and electrochemical impedance spectroscopy, measuring current density and charge transfer resistance, respectively. In addition, surface characterization of AB was performed by scanning electron microscopy, energy-dispersive X-ray spectroscopy, and X-ray photoelectron spectroscopy techniques. Chloroform alkaloid-rich extracts resulted in IE% of 57% at 50 ppm, attributed to the formation of a layer of organic compounds on the AB surface that hindered the dezincification process. The formulation of corrosion inhibitors from *C. lechleri* latex allows for the valorization of non-edible natural sources and the diversification of the offer of green corrosion inhibitors for the chemical treatment of heat exchangers.

## 1. Introduction

Corrosion is the destructive electrochemical attack of a metal by the working environment [1]. The cost of prevention, maintenance, and replacement of damaged infrastructure associated with corrosive processes have been estimated as 5% of an industrialized nation’s gross domestic product (GDP) per year [2]. The design of heat exchangers, which are devices that transfer thermal energy (enthalpy) between two or more fluids [3], requires material with high thermal conductivity and a low coefficient of thermal expansion (CTA). Copper and its alloys are widely used materials for the construction of heat exchangers [4]. Admiralty brass (AB) shows excellent thermal properties (thermal conductivity of 110 W/m K and CTA of 2.02 × 10^−5^ °C^−1^) with a nominal chemical composition of 0.04% As, 71.00% Cu, 1.00% Sn, and 28.00% Zn [5]. However, the negative effects that fouling and corrosion products might have on the copper-based heat exchangers are well-document [6]. This issue can be mitigated by periodic cleaning with acid pickling solutions that contain a corrosion inhibitor to reduce or eliminate the destructive effects of the solution on the metal surface. A corrosion inhibitor is a chemical compound that is added in small concentration to a medium to decrease its corrosive nature [1]. The major industries that use inhibited pickling solutions for maintaining heat exchangers are oil and gas exploration and production, crude oil refining, chemical manufacturing, heavy manufacturing, and water desalination and treatment [7,8]. The corrosion inhibitor market was valued at $3.27 billion worldwide in 2017, with a total consumption of ca. 1.15 million tons [9]. Thiazole, triazole, and tetrazole derivatives are compounds widely employed as a base for the corrosion inhibitors’ formulation used in copper heat exchangers [10,11,12,13,14], but these compounds might have adverse effects on health and the environment [15,16]. Hence, the identification of green alternatives for corrosion inhibitors is required [17]. Natural products have been studied as corrosion inhibitors for which efficiencies are related to their chemical structure, composed in part by heteroatoms with free electron pairs such as O, N, and S capable to bond onto the metal surface [18,19]. Natural extracts that contain alkaloids and phenolic compounds have shown corrosion inhibition for mild steels, carbon steels, aluminum, and cooper in acidic media [20,21,22,23,24,25,26].

*Croton lechleri* is a tree of the family Euphorbiaceae, found in the northwest Amazon basin, especially, in the countries of Ecuador and Peru. This tree produces a deep red latex locally known as “Dragon’s blood”. This latex is used for healing wounds as well as a starting material in the isolation and purification of botanical drugs [27]. The chemical constituents of *C. lechleri* latex are proanthocyanidins, polyphenolic components [28], alkaloids such as tapsine [29], and minor amounts of terpenoid compounds [30]. Both the alkaloids and phenolic compounds contained in *C. lechleri* latex should exhibit inhibition corrosion properties on metallic specimens such as AB in acidic media. The extracts of at least three species of genus *Croton* have been evaluated as corrosion inhibitors with good results under acidic conditions [31,32,33]. 

The extraction of bioactive or functional compounds from vegetable matrices depends on the extraction technique and the solvents used [34]. Lyophilization, solvent and supercritical CO_2_ extraction are techniques employed to obtain high-quality products, with a good process yield, and low environmental impact [35,36,37]. Lyophilization is a concentration method that gently removes water from aqueous extracts [38]. It is performed at low temperature and reduced pressure, which eliminates the risks of heat degradation and bumping. In the case of latex samples, it is adequate for obtaining undamaged mixtures of solved and suspended solids. Soxhlet extraction is a classical extraction method for isolating a compound or a determined group of compounds according to the polarity of the solvent employed [34]. Supercritical fluid extraction with CO_2_ is an alternative non-toxic technique for extracting natural compounds at low temperatures with no use of contaminant solvents [39]. A related technique, named supercritical antisolvent extraction, can assist in overcoming the limited capacity of CO_2_ to dissolve polar compounds [40], resulting in micro- and nanoparticle-sized products [41].

In this study, we used lyophilization, solvent extraction, and supercritical CO_2_ antisolvent extraction to obtain *C. lechleri* solid extracts for their characterization as novel green corrosion inhibitors of AB in hydrochloric acid media. To the best of our knowledge, this is the first report to identify *C. lechleri* extracts as corrosion inhibitors. Phytochemical characterization of the *C. lechleri* extracts showed the presence of alkaloid or phenolic compounds. The corrosion inhibition efficiency (IE%) was electrochemically determined for the three extracts on AB in hydrochloric acid. The corrosion inhibition mechanism of the extract with the highest inhibition efficiency was studied based on electrochemical impedance spectroscopy (EIS), and superficial analyses, such as scanning electron microscopy (SEM), energy-dispersive X-ray spectroscopy (EDS), and X-ray photoelectronic spectroscopy (XPS), indicating the formation of a protective layer of alkaloids on the AB surface.

## 2. Materials and Methods

### 2.1. Lyophilization of C. lechleri Latex (CL1)

A total of 500 mL of Dragon’s blood was purchased from local producers from Tamiahurco village, Napo province, Ecuador. Six samples of 50 mL of *C. lechleri* latex were cooled at −60 °C in an ultra-freezer. Later, they were placed in a lyophilizer (SP Scientific–Genevac, BTP-9E LOVE, Stone Ridge, NY, USA) for five days until constant mass to produce a red-brown solid.

### 2.2. Chloroform Extract from C. lechleri Latex (CL2)

The 100 mL of C. lechleri latex was adjusted to pH 11 with KOH pellets (85%, Fisher Scientific, Pittsburgh, PA, USA). After cooling down the mixture at 3 °C overnight, the resulting red-brown solid was submitted to Soxhlet extraction with 250 mL of chloroform (ACS, EMSURE^®^) for 12 h, twice. Then, the solvent was evaporated under reduced pressure, yielding a beige solid.

### 2.3. Supercritical CO_2_ Antisolvent Extraction from C. lechleri Latex (CL3)

After lyophilization of the *C. lechleri* latex, 30 g of freeze-dried *C.* *Lechleri* underwent dynamic maceration with 500 mL of ethanol at atmospheric pressure and at 20 °C for 19 h. The final concentration of the ethanolic extract was adjusted to 30 mg/mL by vacuum evaporation. Then, CO_2_ flowed continuously at 10 L/min (atmospheric conditions) in a precipitator vessel at supercritical conditions (90 bar, 35 °C). Simultaneously, the ethanolic extract was allowed to flow at 1 mL/min through a 1 mm nozzle. When the supercritical CO_2_ and the ethanolic extract were in contact, an antisolvent extraction occurred (see Figure S1). The solvent and soluble compounds were extracted in the supercritical mixture of CO_2_-ethanol (waste) while the non-soluble compounds precipitated as dry particulate pink solid.

### 2.4. Quantitative Determination of Alkaloids in the Extracts from C. lechleri Latex

Then, 100 mL solutions were prepared with distilled water with concentrations of 1 mg/mL, 0.1 mg/mL and 0.5 mg/mL for CL1, CL2 and CL3, respectively. Sample CL1 was subjected to a previous treatment whereby 10 mL of CL1 solution was adjusted at pH 2 with 2 M HCl followed by rinsing with 3 portions of CHCl_3_ and pH adjusting of the aqueous phase at 7, with 0.1 M NaOH. Then, 5 mL of CL1, CL2 and CL3 solutions were mixed with 5 mL of phosphate-buffered solution at pH 4.7 (prepared with 0.2 M sodium phosphate (96%, Sigma-Aldrich, Burlington, MA, USA)) and 0.2 M citric acid (>99.5%, Sigma-Aldrich) into a separatory funnel. After adding 5 mL of 0.1 mM bromocresol green (95%, Lobal Chemie, Mumbai, India) solution, four portions of CHCl_3_ were placed in the following order: 1 mL, 2 mL, 3 mL, and 4 mL. The resulting mixtures were shaken for 30 min in an automatic shaker. The organic phases were collected and dried over anhydrous magnesium sulphate and filtered. The resulting solution was poured in a 10 mL volumetric flask and the volume was completed with CHCl_3._ The total alkaloid content was determined by spectrophotometry UV-vis (Shimadzu, UV-3600 Plus, Kyoto, Japan) at 416 nm using an atropine (>99%, Sigma-Aldrich, Burlington, MA, USA) standard curve (0–12 µg/mL). Alkaloid values were expressed as atropine equivalents wt% of extract.

### 2.5. Determination of Total Phenols Content in the Extracts from C. lechleri Latex

Additionally, 10 mg of CL extracts were dissolved in 70% MeOH (ACS, EMSURE^®^). An aliquot of 0.5 mL of this solution was poured in a 10 mL volumetric flask. Then, 2 mL of Folin–Ciocalteu reagent (2 M, Sigma-Aldrich, Burlington, MA, USA) were added. The mixture was agitated for 2 min, followed by 5 min repose. Then, 1 mL of 20% Na_2_CO_3_ (>99.5%, Sigma-Aldrich, Burlington, MA, USA) was added, and the volume was completed with 70% MeOH. The resulting mixture was left in the dark for 2 h. Finally, the total phenols content was determined by spectrophotometry UV-vis in the absorbance maximum of gallic acid (97.5–102.5%, Sigma Aldrich) standard curve (0–10 μg/mL). Total phenols values were expressed as gallic acid equivalents wt% of extract.

### 2.6. Electrochemical Determination of the Corrosion Inhibition Efficiency for the Extracts from C. lechleri Latex

Electrochemical measurements were made with potentiostats/galvanostats Autolab PGSTAT128N and Autolab MAC (Metrohm AG, Herisau, Switzerland), employing a three-electrode cell. Experiments were run with Nova 2.1. software. The working electrodes were made of copper admiralty (alloy C44300, nominal chemical composition of 0.0030% Pb, 0.0350% Fe, 0.0430% As, 71.8500% Cu, 1.0300% Sn, and 27.0000% Zn, Metal Samples, Co., Inc., Munford, AL, USA) with an exposed area of 0.283 cm^2^. After the specimen were mechanically ground with 220, 500, 1000, and 2000 emery paper, they were polished with 0.3 µm alumina and washed with ethanol and type I water, then dried, and placed in a cell. A Ag^0^/AgCl/KCl 3 M electrode (CHI111P, CH Instruments, Inc., Bee Cave, TX, USA) and a titanium plate were used as a reference and counter electrodes, respectively. Tafel essays were made by measuring the open circuit potential (OCP) for at least 1800 seconds. Then, a dynamic potential perturbation was applied from −200 mV to 200 mV vs. OCP at a scan rate of 0.5 mV/s. Electrochemical impedance spectroscopy essays were conducted polarizing the working electrode with an alternating signal of 5 mV amplitude, at the OCP, with frequencies from 0.1 Hz to 0.5 MHz. Data were analyzed employing Zview(R) and Origin 8.1. software.

### 2.7. Superficial Characterization of AB Electrodes

All samples were cleaned using compressed air to remove loose particles and other contaminants and placed in a charge-reduction sample holder. SEM micrographs and EDS spectra were measured with a Phenom ProX (Thermo Fisher Scientific, Waltham, MA, USA) with an acceleration voltage of 15.0 kV at 0.5 mbar.

X-ray photoelectron spectroscopy was performed using PHI VersaProbe III (Physical Electronics), equipped with a 180 hemispherical electron energy analyzer, using a monochromatized Al Kα source with energy 1486.6 eV. Energies bandpass of 255 kV and 55 kV were used for Survey and high-resolution operation. The spot size diameter was 100 μm. The polished AB sample was cleaned using a sputtering Ar gun for 3 min to remove carbon and oxidized material.

## 3. Results and Discussion

### 3.1. Phytochemical Screening of the Extracts from C. lechleri Latex

Three different techniques were used to obtain solid extracts from the latex of *C. lechleri* (see Figure S2). Lyophilization of the latex of *C. lechleri* produced a red-brown solid (CL1) with an 18 wt% yield. Chloroform Soxhlet extraction yielded a 0.17 wt% beige solid (CL2), while the supercritical CO_2_ antisolvent extraction gave 1.84 wt% of a pink solid (CL3). Table 1 shows the alkaloid and phenolic compound contents for the three extracts. CL1 and CL3 are phenolic-rich extracts with 46.5 wt% and 52.2 wt%, respectively. CL2 is an alkaloid-rich extract with 51.9 wt% content. Extract CL1 showed both kinds of natural products, while extracts CL2 and CL3 were intentionally enriched in alkaloids and phenolic compounds, respectively. Extract CL2 was obtained after adjusting to alkaline con-ditions (pH = 11), which favored the extraction of alkaloids using chloroform [42]. On the other hand, the sample of the latex of *C. lechleri* was pretreated with ethanol to obtain CL3, which favored the extraction of phenolic compounds [28].

### 3.2. Potentiodynamic Polarization Plots of AB in HCl Media with the Extracts from C. lechleri Latex

**Figure 1:**
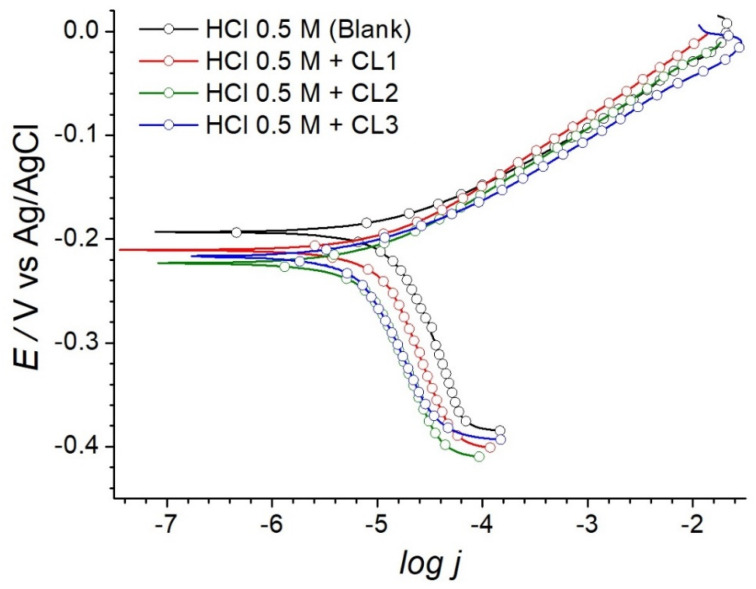
Potentiodynamic polarization plots of AB in 0.5 M HCl in the presence of various extracts of extracts from *C. lechleri* latex at 25 °C.

Figure 1 shows the potentiodynamic polarization curves for AB in 0.5 M hydrochloric acid aqueous solution at 25 °C employing the three different extracts from *C. lechleri* latex CL1, CL2, and CL3 at a concentration of 300 ppm, 50 ppm, and 200 ppm, respectively. The solubility limit was reached for the three extracts under the working conditions. In all cases, the corrosion current density decreased with the presence of the extracts, which indicates the corrosion inhibition on the surface of AB. These *C. lechleri* extracts could be classified as a mixed-type inhibitor because of a remarkable decrease in the cathodic as well as corrosion currents, with a slight decrease in the anodic currents and shift in the corrosion potential (E_corr_) to lower values [43,44]. This effect significantly increased with increased concentrations of CL3 from 50 ppm to 200 ppm (see Figure S3).

Electrochemical parameters, including E_corr_, cathodic Tafel slope (β_c_), anodic Tafel slope (β_a_), corrosion current density (j_corr_), and IE% are provided in Table 2. The j_corr_ and E_corr_ were obtained from the extrapolation of anodic and cathodic Tafel linearized current regions. Values of IE% were determined using Equation (1) as follows:

(1) $$\mathrm{IE}\%=\left(1-\frac{j_{\mathrm{corr}}}{j_{\mathrm{corr}}^{0}}\right)\times 100$$

where $j_{\mathrm{corr}}^{0}$ and j_corr_ represent the corrosion current densities in the absence and presence of inhibitor, respectively. CL1 inhibited in 30.48% the corrosion of AB in HCl media. The CL3 sample showed an improved inhibition of 48.93% compared to CL1, despite its lower concentration. This result might be related to a higher phenolics content and compound purification due to the supercritical antisolvent process [45]. Moreover, the size and the spheric shape of the particles obtained in CL3, which showed diameters of about 300–600 nm (see Figure S4), might increase the surface coverage area and availability compared to the coarse CL1 powder [46,47,48]. The IE% was found to increase continuously with an increase in the concentration of CL3 (see Table S2). The highest inhibition efficiency of 51.57% was reached with the alkaloid-rich CL2 in a low concentration of 50 ppm. The use of biomolecules for the formulation of corrosion inhibitors is expected to have a lower environmental impact compared to commercial inhibitors from chemical synthesis [49]. Hereafter, we focused on CL2 for further analysis to develop better insight into the inhibition process.

### 3.3. EIS of AB in HCl Media Inhibited with CL2

**Figure 2:**
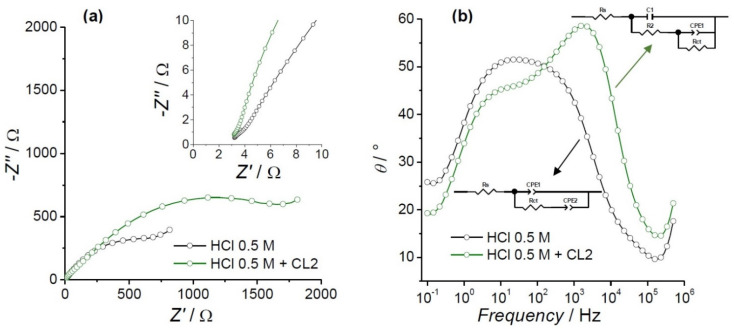
Electrochemical impedance spectroscopy diagrams of AB at E_corr_ in 0.5 M HCl, in the absence (black lines) and the presence (green lines) of CL2: (**a**) Nyquist plots (inset: zoom in at high frequencies) and (**b**) Bode diagrams (insets: equivalent circuits).

After Tafel measurements, EIS was carried out by varying the frequency from 0.1 Hz to 0.5 MHz. Equation (2) was used to calculate IE% as follows:

(2) $$\mathrm{IE}\%=\left(1-\frac{R_{\mathrm{ct}}^{0}}{R_{\mathrm{ct}}}\right)\times 100$$

where $R_{\mathrm{ct}}^{0}$ and R_ct_ represent the charge transfer resistance in the absence and presence of CL2, respectively. Figure 2 shows the impedance diagrams obtained in the presence and absence of 50 ppm of CL2 at E_corr_ in HCl 0.5 M. The Nyquist plot reveals one loop at high frequencies (see Figure 2a inset). A second loop is observed at middle frequencies and the beginning of a diffusional impedance for both spectra (see Figure 2a). All loops are distorted semicircles. Deviations of the ideal semicircles can be attributed to the inhomogeneities on the alloy surface such as point defects, grain boundaries, and lattice distortions [50]. Independent of the polishing treatment, the admiralty surface formed oxide films by the contact of the metallic electrode with atmospheric or dissolved oxygen. This thin oxide layer is observed in the Nyquist plot as one loop at high frequencies.

The Nyquist spectra with and without the inhibitor were fitted using equivalent circuits (see Figure 2b insets), where R_s_ is the solution resistance, R_ct_ is the charge transfer resistance, CPE1 and CPE2 are constant phase elements, R2 is the oxide film resistance, and C1 the capacitance for the oxide film. EIS fitting parameters are summarized in Table 3. For AB in HCl media, a CPE1 of 496.6 (µF)^n^ was obtained with a value of n = 0.63, describing the behavior of the capacitor, while a value of 6.29 (mF)^n^ was determined with n = 1.13 for the CPE2. For the impedance spectra, fitting in the presence of CL2, C1 was found to have a value of 3.45 µF. This circuit is composed of two-time constants nested, which indicate a porous or inhomogeneous layer onto the AB where the charge transfer occurs (see Figure 2b) [12]. Similar behavior was observed in a set of trials performed with different concentrations of CL3 (see Figure S5), where a new time constant emerged as the CL3 concentration increased. The increase of R_ct_ in the presence of CL2 is due to the formation of a layer of inhibitor that prevents electronic interchange. An IE% of 57.15% was determined using Equation (2), comparable to the one determined by Tafel plots.

### 3.4. Investigation of the Morphology and Surperficial Composition of AB by SEM-EDS and XPS

**Figure 3:**
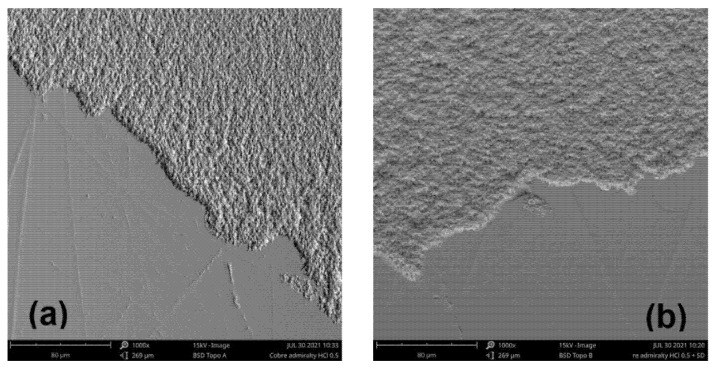
SEM micrographs of AB (**a**) in 0.5 M HCl and (**b**) in 0.5 M HCl with 50 ppm CL2.

Figure 3 shows the SEM images of AB after immersion in 0.5 M HCl solutions in the absence and presence of CL2 at 50 ppm. The surface of the metal sample was significantly damaged in the HCl solution, indicating a high level of corrosion (see Figure 3a), while in the presence of CL2, the surface of AB was smoother, indicating a decreased corrosion rate (see Figure 3b). This phenomenon might be related to the formation of a stable protective layer of CL2 on the AB surface.

**Figure 4:**
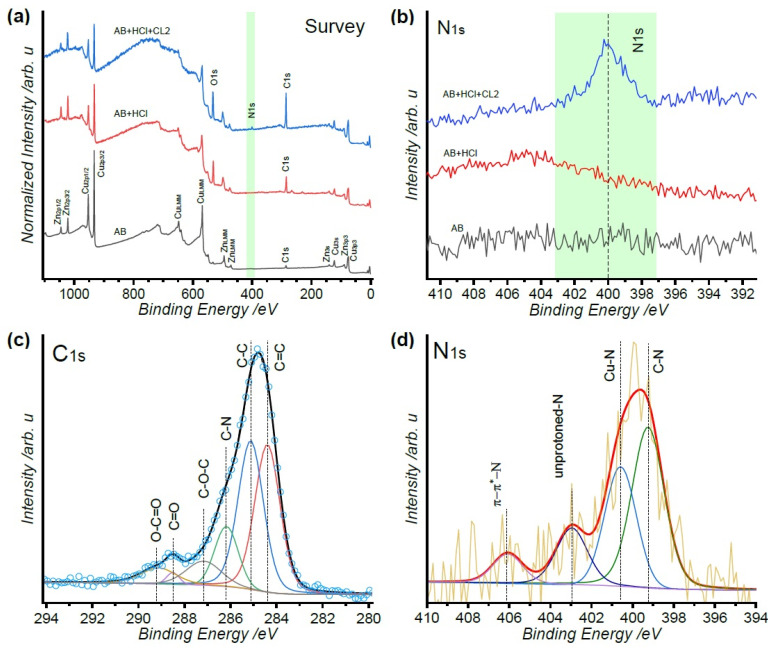
XPS spectra of the (**a**) survey scan and (**b**) N1s for a polished AB (black line), AB after immersion in 0.5 M HCl (red line), and AB after immersion in 0.5 M HCl with 50 ppm CL2 (blue line). (**c**) C1s and (**d**) N1s spectra for the CL2 inhibited AB sample.

Comparative XPS survey spectra for a polished metal sample (black line), AB immersed in 0.5 M HCl (red line), and AB immersed in 0.5 M HCl with 50 ppm CL2 (blue line) are shown in Figure 4a. The inhibited-metal sample presented increased oxygen and carbon peaks, which can be mainly attributed to the organic composition of alkaloid-rich CL2 deposition on the surface. Moreover, nitrogen was only observed in the AB surface treated in the presence of CL2 confirming the presence of an alkaloid-rich layer in the surface (see Figure 4b). This layer attenuated the metallic register intensity of copper and zinc. As shown in Figure 4c, the C1s core level presented several components such as C=C binding at 284.4 eV and C-C at 285.12 eV. A shoulder was attributed to C-N at 286.17 eV and three additional peaks at 287.16 eV, 288.5 eV, and 289.12 eV associated with C-O-C, C=O, and O-C=O, respectively [51,52]. These types of bonds can be found in the tapsine alkaloid (see Figure S6), which has been isolated from the latex of *C. lechleri* [29]. The N1s high-resolution feature presents chemical environments for C-N and Cu-N binding energies at 400.45 eV and 399.35 eV, respectively, an unprotonated N atom binding at 402.75 eV followed by π-π * plasmon at 406.06 eV, as presented in Figure 4d [53,54,55]. Thus, the alkaloid-rich extract CL2 can form a protective film on the AB/solution interface via the Cu-N coordination bonds through N lone pair and copper. This barrier film effectively hinders the corrosion of the AB substrate by the HCl medium. The Cu2p3 feature presented a typical orbital splitting with an ∆E = 19.9 eV for all the samples (see Figure S7a). The full width at half maximum (FWHM) changed from 1.293 eV for the polished metal sample to 1.414 eV for the corroded samples related to the formation of Cu(I). The latter was confirmed by the weak satellites at 947 eV. The behavior of Zn2p3 presented a splitting orbital with an increment of the FWHM from 1.479 eV for the polished AB to 1.863 eV for the corroded samples as well as a shift on the Zn2p3/2 peak of 0.394 eV, which was attributed to the formation of Zn(II) on the surface (see Figure S7b).

The mechanism of brass corrosion in acidic media has been reported as a two-step dezincification process [56]. Initially, zinc is preferentially or even selectively dissolved [57] followed by a dissolution–redeposition of copper on the alloy surface, generating copper-rich islands or porous films [58] as follows:

(3) $$\mathrm{Cu}-\mathrm{Zn}\to {\mathrm{Zn}}^{2+}+2e^{-}+\mathrm{Cu}$$

(4) $$\mathrm{Cu}\to {\mathrm{Cu}}^{+}+e^{-}$$

(5) $$2{\mathrm{Cu}}^{+}+\mathrm{Zn}\to 2\mathrm{Cu}+{\mathrm{Zn}}^{2+}$$

Our experimental results agree with this process, as evidenced by the difference in the surface composition of Cu (70.48%) and Zn (29.42%) for a polished metal sample (see Figure S8) compared to the composition of Cu (82.12%) and Zn (17.88%) for AB treated in 0.5 M HCl (see Figure S9). The inhibition capacity of CL2 is attributed to the molecular structure of natural products that were enriched in alkaloid compounds. Tapsine structure shows two aromatic rings with π electrons and heteroatoms such as nitrogen and oxygen, that can interact with the d-orbitals of the metal by their lone pairs to form a well-adsorbed organic film. The layer, formed on the surface, can isolate the alloy from the corrosive solution, which has been observed in mixed-type inhibitors [59,60,61,62] as evidenced by the conserved surface composition of Cu (69.70%) and Zn (30.30%) for AB treated in 0.5 M HCl with 50 ppm CL2 (see Figure S10). Furthermore, many alkaloids as well as other natural products form complexes with Cu+ or Zn^2+^ that prevents the formation of soluble metallic chlorides, limiting the overall oxidation process [63]. A comparison of the AB samples by the naked eye after immersion in 0.5 M HCl solution in the absence and presence of CL2 at 50 ppm showed the inhibition potential of dragon’s blood extracts (see Figure S11). From an industrial point of view, the formulation of corrosion inhibitors based on *C. lechleri* latex offers two opportunities—the valorization of a non-edible natural source and the diversification of the availability of corrosion inhibitors for the chemical treatment of equipment in different industrial sectors. These green corrosion inhibitors might provide an option for the corrosion mitigation in the cleaning process of heat exchangers increasing their lifecycle.

## 4. Conclusions

In summary, lyophilization, chloroform Soxhlet extraction, and supercritical CO_2_ antisolvent extraction techniques were successfully employed to obtain novel green *C. lechleri* solid corrosion inhibitors of AB in 0.5 M HCl media. CL1, which is the dry *C. lechleri* latex, showed the presence of alkaloid and phenolic compounds with an IE% of 30.48%. Microparticles of CL3 enriched in phenolic content provided an IE% of 48.93%, while CL2 was enriched in alkaloid compounds showing an IE% of 57%. Adsorption onto the surface of AB of the alkaloid molecules in CL2 was proposed as the mechanism of corrosion by slowing down the dezincification process of the metal sample. Further purification and processing of CL2 should improve its solubility and corrosion-inhibition efficiency. Studies at various concentrations and temperatures as well as computational simulations are performing by our group to obtain information about the adsorption process and thermodynamics.

## Figures and Tables

**Table 1:** Alkaloids and phenolic compounds content for triplicated measurements of the solid extracts obtained from the *C. lechleri* latex.

Entry	Alkaloid Content (wt. %)	Phenolic Compound Content (wt. %)
CL1	2.1 ± 0.1	46.5 ± 1.9
CL2	51.9 ± 1.2	3.7 ± 0.2
CL3	0.7 ± 0.4	52.2 ± 0.4

**Table 2:** Tafel polarization parameters for AB in 0.5 M HCl with extracts from *C. lechleri* latex at 25 °C.

Entry	E_corr_ (V)	β_c_ (mV/dec)	β_a_ (mV/dec)	j_corr_ (μA/cm^2^)	IE%
HCl 0.5 M	−0.193	281.6	53.8	14.84	-
HCl 0.5 M + CL1	−0.210	261.5	62.9	10.32	30.48
HCl 0.5 M + CL2	−0.211	241.5	57.5	7.19	51.57
HCl 0.5 M + CL3	−0.214	238.0	47.5	7.58	48.93

**Table 3:** Results of resistance and goodness fit values from impedance measurements.

Entry	R_s_	R_ct_	R_1_	Sum of Squares	IE%
HCl 0.5 M	3.049	1221	-	0.03649	-
HCl 0.5 M + CL2	3.602	2850	19.83	0.24907	57.16

## Data Availability

Data are available from the authors upon request.

## Supplementary Materials

The following are available online: Figure S1. Pilot plant of supercritical CO_2_ antisolvent extraction of CL3. Table S1. Conditions of supercritical CO_2_ antisolvent extraction of CL3. Figure S2. Photographs of solid extracts obtained from *C. lechleri* by lyophilization, solvent extraction, and supercritical CO_2_ antisolvent extraction. Figure S3. Potentiodynamic polarization plots of AB in 0.5 M HCl in the presence of CL3 at several concentrations at 25 °C. Figure S4. Microphotography of CL3 spherical particles. Table S2. Tafel polarization parameters for AB in 0.5 M HCl with CL3 at concentration of 50 ppm–200 ppm at 25 °C. Figure S5. Bode diagrams of AB at E_corr_ in 0.5 M HCl at various concentrations of CL3. Figure S6. Chemical structure of tapsine. Figure S7. Cu2p3 and Zn2p3 XPS spectra for a polished AB, AB after immersion in 0.5 M HCl, and AB after immersion in 0.5 M HCl with 50 ppm CL2. Figure S8. EDS spectrum of a polished AB sample. Figure S9. EDS spectrum of AB after immersion in 0.5 M HCl. Figure S10. EDS spectrum of AB after immersion in 0.5 M HCl with 50 ppm CL2. Figure S11. Photographs of AB after immersion in 0.5 M HCl and 0.5 M HCl with 50 ppm CL2.

## Sample Availability

Samples of the compounds are available from the authors.
