# Supplementary material for: Croton lechleri Extracts as Green Corrosion Inhibitors of Admiralty Brass in Hydrochloric Acid
Source: Molecules. 2021 Dec 7;26(24):7417. doi: 10.3390/molecules26247417 (PMC8706740; doi:10.3390/molecules26247417)
Supplement: Supplementary file 1 [file molecules-26-07417-s001.zip › molecules-1490455-supplementary.pdf]

SUPPLEMENTARY MATERIALS

Croton Lechleri Extracts as Green Corrosion Inhibitors of Admiralty Brass in Hydrochloric Acid

Carlos Cevallos-Morillo [1], Pablo Cisneros-Pérez [2], Roxana Llive [2], Marvin Ricaurte [3], Carlos Reinoso [4], Miguel Angel Meneses [5], Maria del Cisne Guamán [5], and Alex Palma-Cando [3,*]

[1] Facultad de Ciencias Químicas, Universidad Central del Ecuador, Francisco Viteri s/n y Gato Sobral, 170129 Quito, Ecuador; cacevallosm@uce.edu.ec

[2] Universidad Regional Amazónica Ikiam, Tena, Napo, Ecuador; pablo.cisneros@ikiam.edu.ec (P.C.-P.); Roxana.llive@ikiam.edu.ec. (R.L.)

[3] Yachay Tech University, School of Chemical Sciences and Engineering, Grupo de Investigación Aplicada en Materiales y Procesos (GIAMP), Hda. San José s/n y Proyecto Yachay, 100119 Urcuquí, Ecuador; mricaurte@yachaytech.edu.ec (M.R.); apalma@yachaytech.edu.ec (A.P.-C.)

[4] Yachay Tech University, School of Physical Sciences and Nanotechnology, Hda. San José s/n y Proyecto Yachay, 100119 Urcuquí, Ecuador; creinoso@yachaytech.edu.ec (C.R.)

[5] Departamento de Química y Ciencias Exactas, Universidad Técnica Particular de Loja, Loja 110150, Ecuador; mameneses@utpl.edu.ec (M.A.M.); mcguaman@utpl.edu.ec (M.C.G.-B.)

* Correspondence: apalma@yachaytech.edu.ec

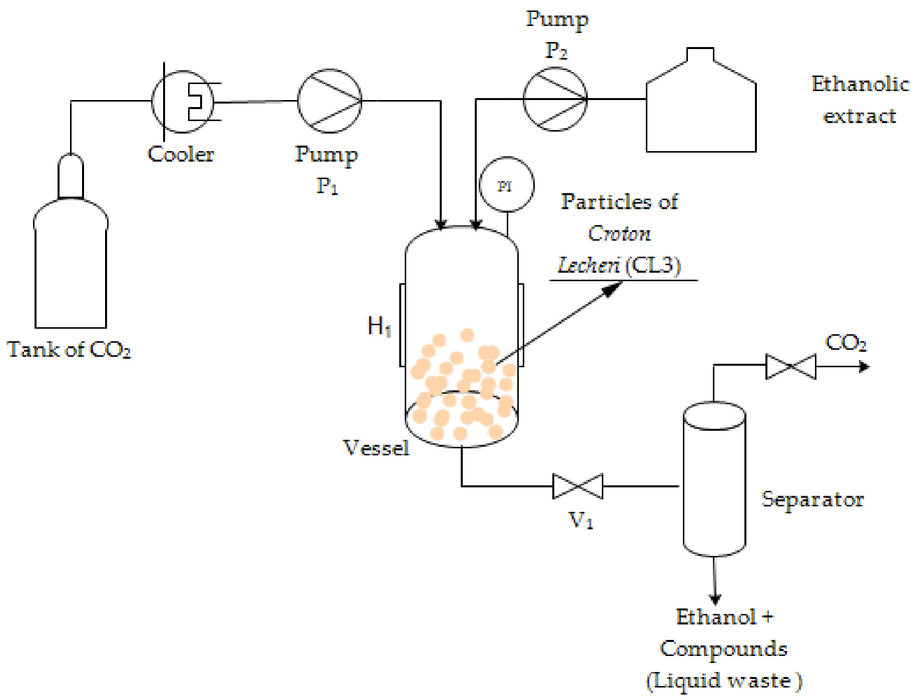

Figure S1. Pilot plant of supercritical CO_2 antisolvent extraction of CL3.

A home-made pilot plant was used in this study, the schematic diagram is shown in Figure S9. The equipment consisted in one tank of CO_2 and a cooler, two pumps, one for CO_2 (P1) and the other for the liquid extract (P2). These two flow currents mixed at a heated jacketed (H1) 250 mL precipitator vessel connected to a pressure gauge (PI). A micrometric valve (V1) allowed to control flux to the separator where CO_2 (g) was released from the ethanolic waste mixture. The values of the parameters used are shown in Table S1.

Table S1. Conditions of supercritical CO_2 antisolvent extraction of CL3.

**Pressure (bar)**	**Temperature (°C)**	**Concentration of the extract (mg/mL)**	**Flow of CO_2 (mL/min)**	**Time of wash (min)**
90	35	30	10000	60

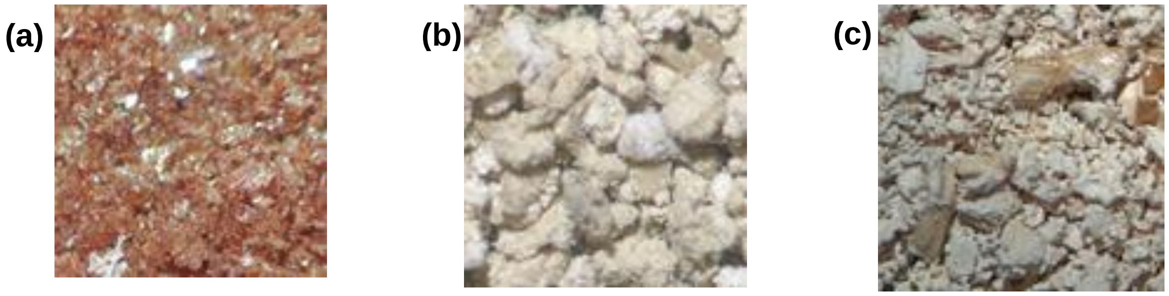

Figure S2. Photographs of solid extracts obtained from *C. lecheri* by (a) lyophilization, (b) solvent extraction, and (c) supercritical CO_2 antisolvent extraction.

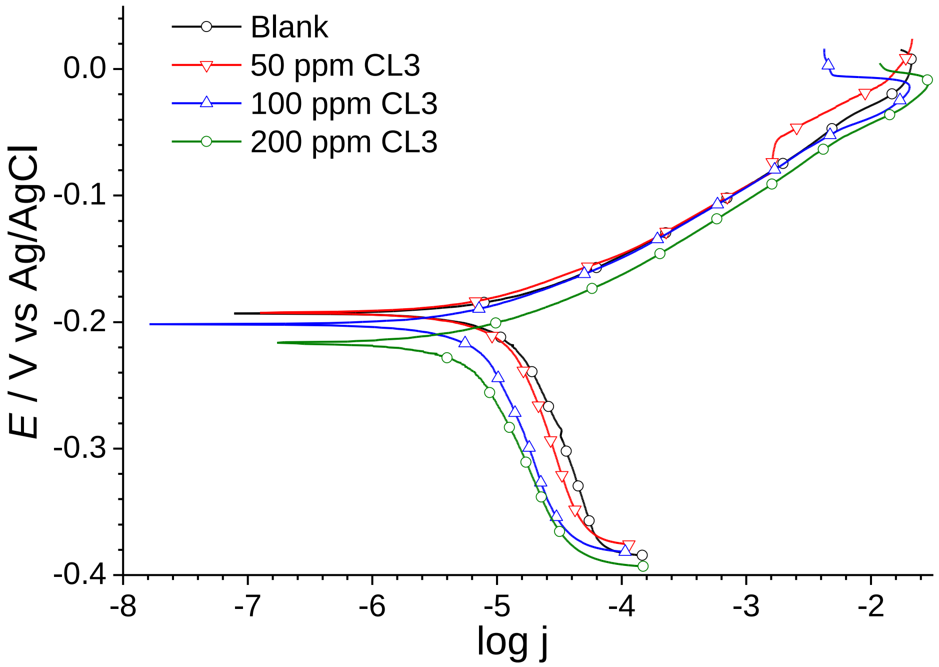

Figure S3. Potentiodynamic polarization plots of AB in 0.5 M HCl in the presence of CL3 at several concentrations at 25°C.

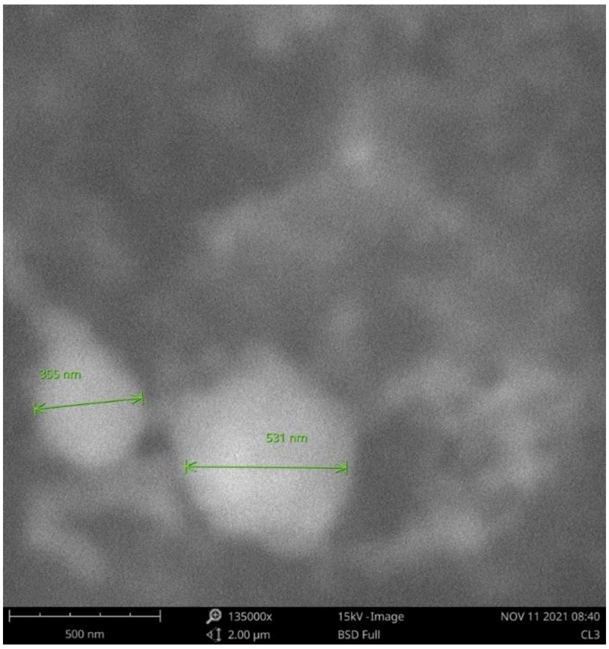

Figure S4. Microphotography of CL3 spherical particles.

Table S2. Tafel polarization parameters for AB in 0.5 M HCl with CL3 at concentrations of 50 ppm-200 ppm at 25°C.

Entry		E_{corr} (V)	β_c (mV/dec)	β_a (mV/dec)	j_{corr} (µA/cm^2)	IE%
Blank		-0.193	281.6	53.8	14.84	-
	50 ppm	-0.194	360.5	53.1	13.13	11.56
CL2	100 ppm	-0.202	308.5	45.5	8.95	39.72
	200 ppm	-0.214	238.0	47.5	7.58	48.93

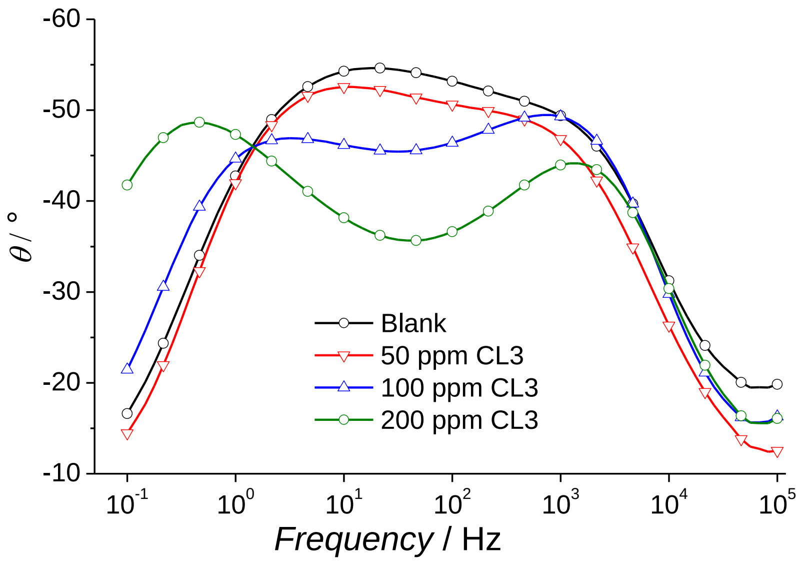

Figure S5. Bode diagrams of AB at E_{corr} in 0.5 M HCl at various concentrations of CL3.

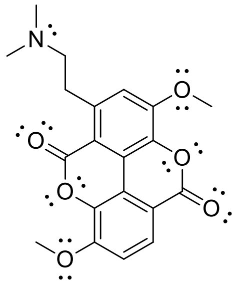

Figure S6. Chemical structure of tapsine.

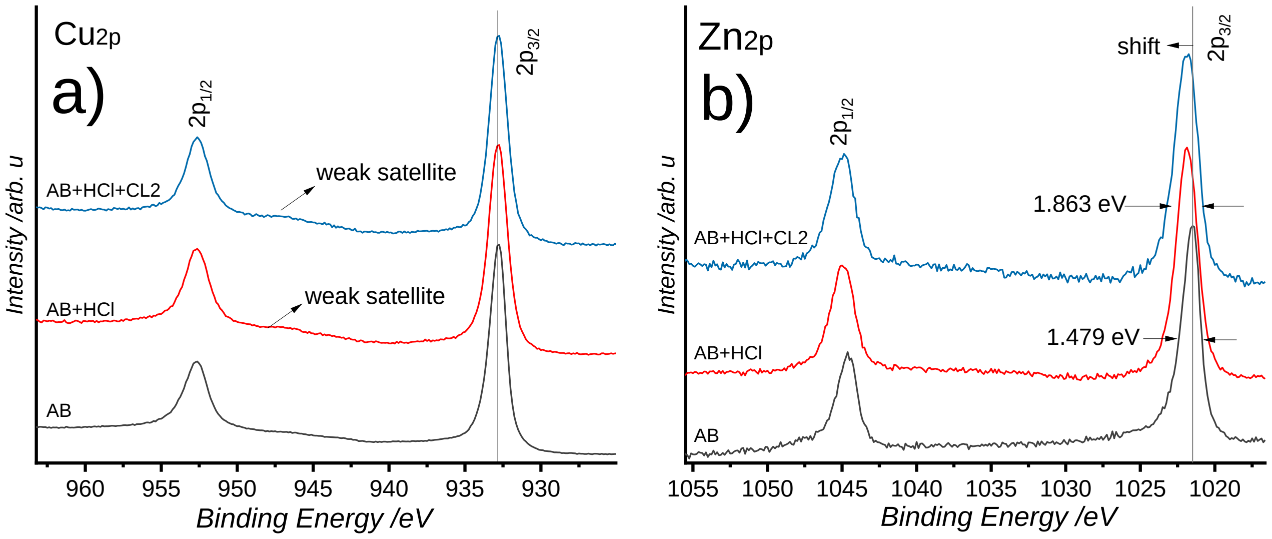

Figure S7. (a) Cu2p3 and (b) Zn2p3 XPS spectra for a polished admiralty brass (black line), admiralty brass after immersion in 0.5 M HCl (red line), and admiralty brass after immersion in 0.5 M HCl with 50 ppm CL2 (blue line).

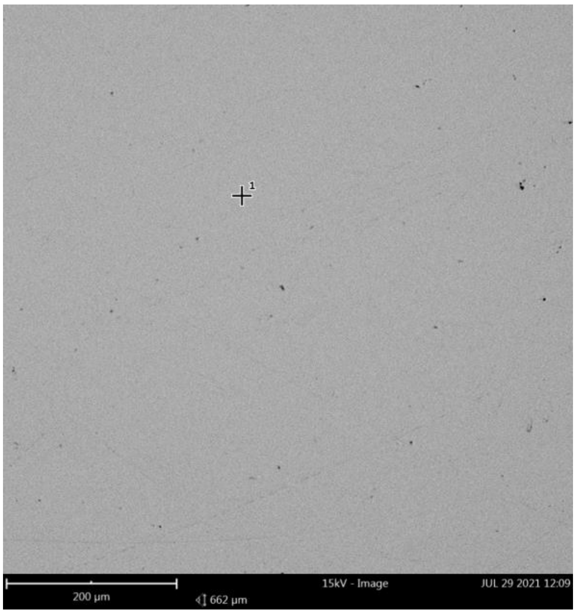

Element Symbol	Atomic Conc.	Weight Conc.	Oxide Symbol	Stoich. Conc.
Cu	70.58	69.99	Cu	70.58
Zn	29.42	30.01	Zn	29.42

FOV: 662 µm, Mode: 15kV - Image, Detector: BSD Full

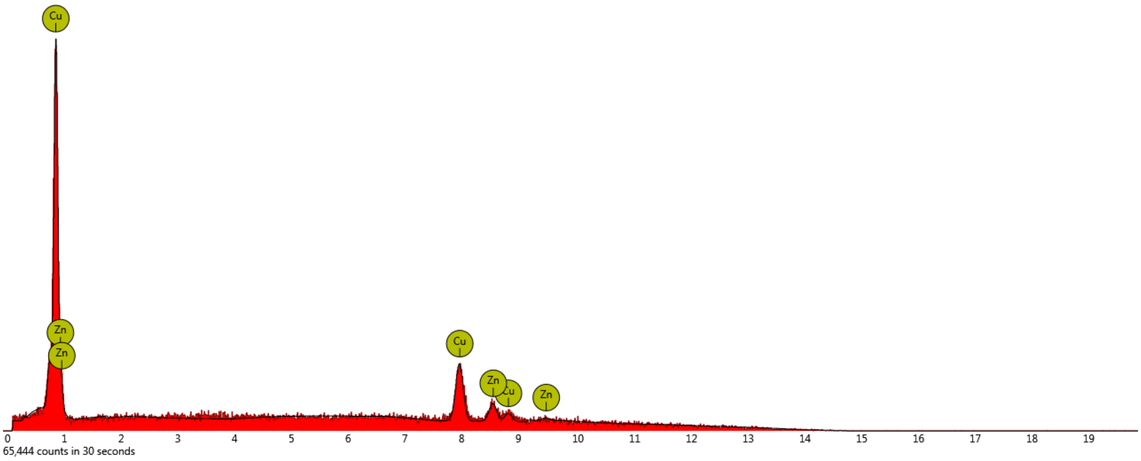

Figure S8. EDS spectrum of a polished admiralty brass sample.

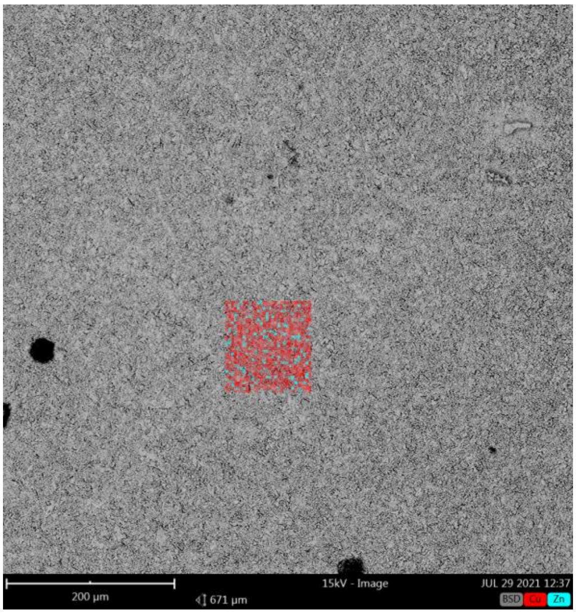

Element Symbol	Atomic Conc.	Weight Conc.	Oxide Symbol	Stoich. Conc.
Cu	82.12	81.70	Cu	82.12
Zn	17.88	18.30	Zn	17.88

FOV: 671 µm, Mode: 15kV - Image, Detector: BSD Full

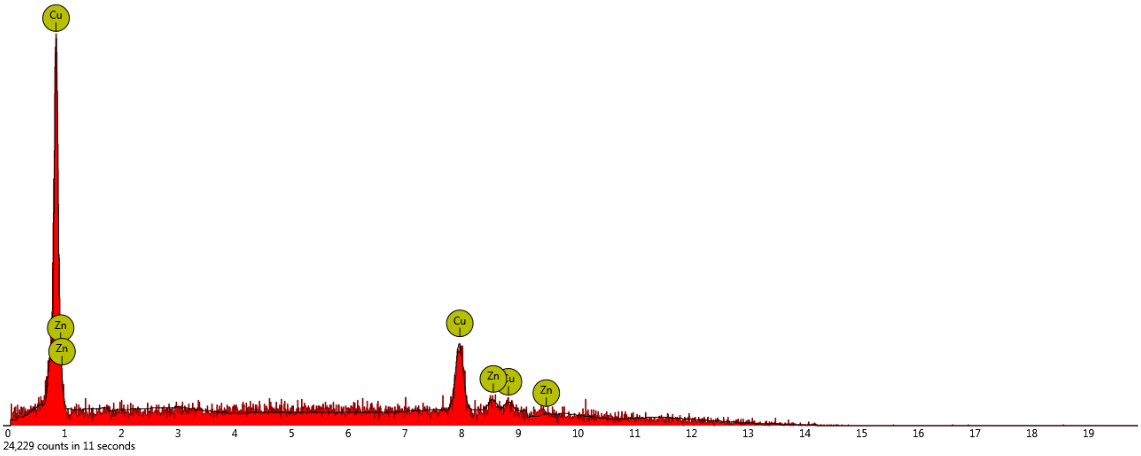

Figure S9. EDS spectrum of admiralty brass after immersion in 0.5 M HCl.

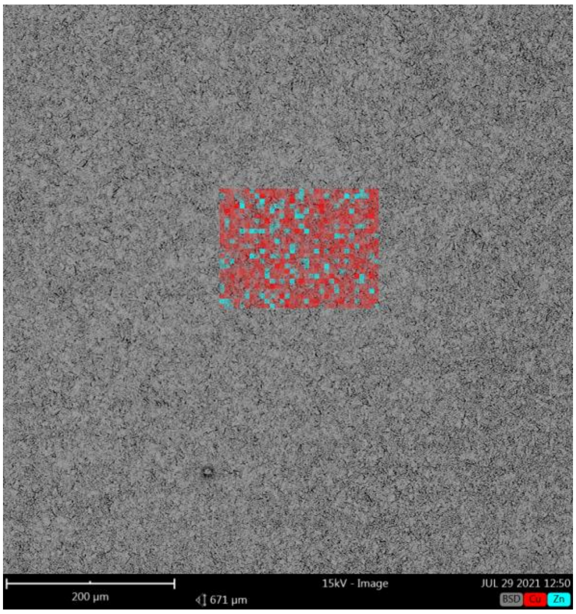

Element Symbol	Atomic Conc.	Weight Conc.	Oxide Symbol	Stoich. Conc.
Cu	69.70	69.10	Cu	69.70
Zn	30.30	30.90	Zn	30.30

FOV: 671 µm, Mode: 15kV - Image, Detector: BSD Full

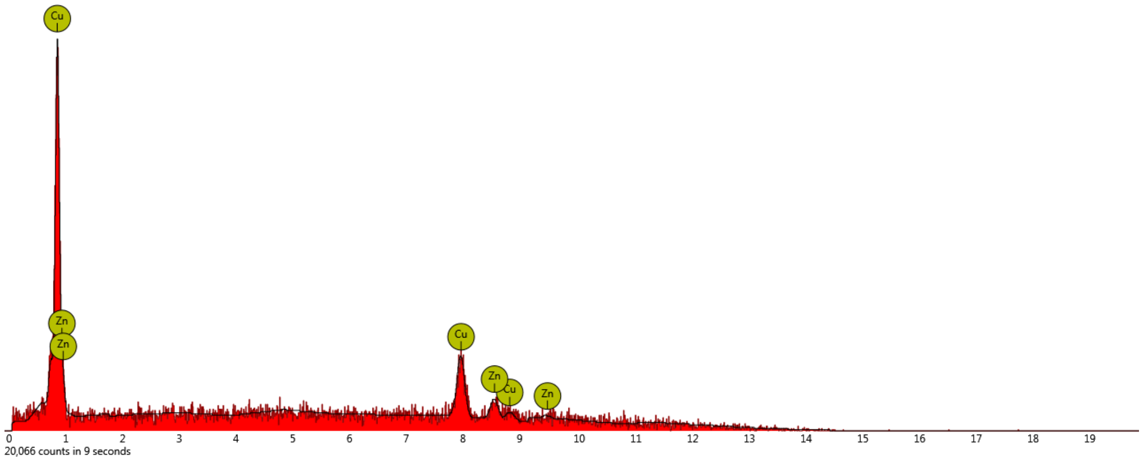

Figure S10. EDS spectrum of admiralty brass after immersion in 0.5 M HCl with 50 ppm CL2.

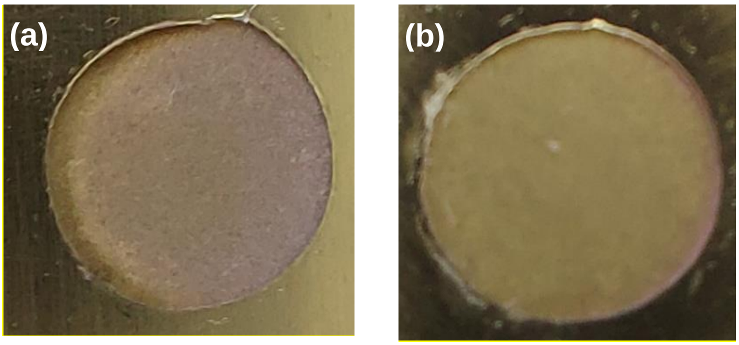

Figure S11. Photographs of admiralty brass after immersion in (a) 0.5 M HCl and (b) 0.5 M HCl with 50 ppm CL2.
